# Nasal carriage of a single clone of community-acquired methicillin-resistant S*taphylococcus aureus *among kindergarten attendees in northern Taiwan

**DOI:** 10.1186/1471-2334-7-51

**Published:** 2007-06-01

**Authors:** Wen-Tsung Lo, Wei-Jen Lin, Min-Hua Tseng, Jang-Jih Lu, Shih-Yi Lee, Mong-Ling Chu, Chih-Chien Wang

**Affiliations:** 1 Department of Pediatrics, Tri-Service General Hospital, National Defense Medical Center, Taipei, Taiwan.; 2 Graduate Institute of Medical Sciences, National Defense Medical Center, Taipei, Taiwan.; 3 Department of Pathology, Tri-Service General Hospital, National Defense Medical Center, Taipei, Taiwan.

## Abstract

**Background:**

To evaluate the prevalence and microbiological characterization of community-acquired (CA) methicillin-resistant *Staphylococcus aureus *(MRSA) nasal carriage in a kindergarten.

**Methods:**

Point prevalence study. Nasal swabs were collected from healthy children younger than 7 years of age who were attending a kindergarten in Taipei, Taiwan. A parent questionnaire regarding MRSA risk factors was administered simultaneously. All CA-MRSA colonization isolates were archived for subsequent antimicrobial susceptibility and molecular typing.

**Results:**

Of the 68 children who participated in the study, 17 (25%) had *S. aureus *isolated from nasal swabs. Nine (13.2%) of the 68 children had CA-MRSA carriage, and none of them had any identified risk factors. Antimicrobial susceptibility testing revealed all of the 9 CA-MRSA colonization isolates had uniformly high resistance (100%) to both clindamycin and erythromycin, the macrolide-lincosamide-streptogramin-constitutive phenotype and the *ermB *gene. Pulsed-field gel electrophoresis revealed 8 (88.9%) of 9 CA-MRSA colonization isolates were genetically related and multilocus sequence typing revealed all isolates had sequence type 59. All of the colonization isolates carried the staphylococcal cassette chromosome *mec *type IV, but none were positive for the Panton-Valentine leukocidin genes.

**Conclusion:**

The results of this study suggest that a single predominant CA-MRSA colonization strain featuring high clindamycin resistance circulated in this kindergarten. Additionally, due to the established transmissibility of colonization isolates, the high prevalence of nasal carriage of CA-MRSA among healthy attendees in kindergartens may indicate the accelerated spread of CA-MRSA in the community.

## Background

Beginning in the late 1990s, studies from various cities in the United States and other countries reported a significant prevalence of community-acquired (CA) methicillin-resistant *Staphylococcus aureus *(MRSA) colonization or infection among both children and adults [1-6], and especially the presence of increasing numbers of patients with CA-MRSA who do not appear to demonstrate evident risk factors [2,4-6]. Reports of pediatric deaths as a consequence of CA-MRSA infections further illustrated the potential seriousness of this emergence [1].

Recently, the results of our epidemiological investigation relating to CA-MRSA infections among healthy children in Taiwan have also been a cause for concern because they indirectly reflect that there might be a reservoir of children with asymptomatic CA-MRSA colonization in the community [7]. Because of the geographic diversity in the prevalence of MRSA carriage and the possible transmission from any individual colonized with MRSA [8], measurement of the rate of CA-MRSA carriage may be helpful to estimate the potential for spread of CA-MRSA in the community. Therefore, this preliminary study was conducted to determine the prevalence of CA-MRSA colonization of the anterior nares among healthy attendees of a kindergarten. In addition to characterizing these CA-MRSA colonization isolates, we also compared them with healthcare-associated MRSA (HA-MRSA) isolates via molecular analyses.

## Methods

### Study subjects

This was a point prevalence study conducted in a kindergarten in northern Taiwan. The study proposal was reviewed and approved by the National Defense Medical Center Institutional Review Board. Participant or parental informed consent was obtained in all cases. The kindergarten was a for-profit facility that the director had owned and operated for 28 years. Attendees were divided into the following four classes by age: toddler (2-younger than 4 years), older toddler (4-younger than 5 years), preschool child (5-younger than 6 years), and child attending the kindergarten prior to leaving for school (6-younger than 7 years). All classes shared a room for an average of one-half hour in the morning and indoor play areas.

All kindergarten attendees younger than 7 years of age, regardless of their medical history, were considered eligible to participate in this study. Participants and their guardians were approached by the same investigative team throughout the study period.

### Cultures and questionnaires

After obtaining written consent, study personnel verbally administered a questionnaire to the guardians to collect demographic data and information on risk factors of all children and their household contacts. Risk factors for MRSA infection analyzed in this study included the following: (1) hospitalization, surgery, endotracheal intubation or antimicrobial therapy in the previous 12 months, (2) underlying chronic disorder (e.g., asthma, chronic lung disease, atopic dermatitis, heart disease or neurological disease) (3) presence of an indwelling venous or urinary catheter, or (4) household contact with an individual with an identified risk factor, (e.g., long-term care facility residence, intravenous drug abuse, recurrent skin infections, history of MRSA infection or colonization) or a worker in a health care environment in the 12 months preceding the culture [9].

A specimen for culture was obtained from both anterior nares of each enrolled child with a sterile dry cotton swab, pre-moistened with sterile water. The swab was immediately inoculated onto 5% sheep blood agar which was then incubated for 36 to 48 hours at 35°C.

### Bacterial strains and antimicrobial susceptibility testing

Staphylococci were identified based on colonial morphology, catalase testing, tube-coagulase testing, DNase reaction, mannitol fermentation, tellurite reduction, and an oxidation-fermentation test. MRSA identification and antimicrobial susceptibility were determined according to the Clinical Laboratory Standards Institute (formerly known as the National Committee for Clinical Laboratory Standards) guidelines [10,11]. In vitro macrolide-lincosamide-streptogramin-inducible (MLS_i_) phenotypes were detected by the double-disk diffusion assay [12]. During the study period, all children colonized with CA-MRSA isolates were compared with consecutive control subjects with HA-MRSA infection. Susceptibility to penicillin-G, oxacillin, clindamycin, erythromycin, gentamicin, vancomycin, tetracycline, ciprofloxacin and trimethoprim/sulfamethoxazole was determined using the disc-diffusion method [10,11].

### Multilocus sequence typing (MLST)

MLST was performed by polymerase chain reaction (PCR) amplification and sequencing of seven housekeeping genes using primers designed by Enright et al [13]. Each sequence was submitted to the MLST database website for assignment of the allelic profile and sequence type (ST).

### Pulsed-field gel electrophoresis (PFGE)

All *S. aureus *nasal-colonization isolates were analyzed for epidemiologic relatedness by PFGE of chromosomal DNA performed using the enzyme *Sma*I (New England Biolabs, Beverly, Mass, USA). DNA was separated in 0.9% agarose gels at 14°C in 0.5× TBE buffer with a CHEF Mapper XA system (Bio-Rad Laboratories, Hercules, CA, USA) for 31.5 h, with initial and final switching times of 2 and 30 s, respectively. Gels were stained with ethidium bromide and photographed under UV illumination. The derived patterns were analyzed using GelCompar software (Applied Maths, Kortrijk, Belgium). Results were analyzed using the unweighted pair group method for arithmetic averages (UPGMA) and the Dice coefficient with 1.2% band tolerance [14]. The strain types were designated in alphabetical order, and a new type, if identified, was designated consecutively. MRSA isolates sharing closely related PFGE profiles from one existing strain type were defined as its subtypes and labeled with suffixes of Arabic numbers.

### Staphylococcal cassette chromosome *mec* (SCC*mec*) typing

SCC*mec *typing was performed by means of PCR using sets of region-specific primers as described elsewhere [15,16].

### PCR amplification of *mecA*, *lukS-PV*, *lukF-PV*, *ermA*, *ermB*, *ermC* and *msrA*

PCR for *mecA *was performed using relevant published sequences and temperature parameters [17]. The PCR amplification of the *lukS-PV*, *lukF-PV*, and genes encoding Panton-Valentine leukocidin (PVL) components was performed as described elsewhere [18]. The presence of MLS resistance genes (*ermA*, *ermB, ermC *and *msrA*) was determined according to previously described methods [19,20].

### Statistical analysis

Data collection and analyses were performed with the Fisher's exact test using SPSS software, version 10.0 (Statistical Package for Social Sciences; SPSS for Windows, Inc., Chicago, IL, USA). A *P *value of < 0.05 was considered to represent a statistically significant difference between tested groups.

## Results

### Prevalence of *S. aureus* and CA-MRSA nasal carriage

Sixty-eight children with ages ranging from 2.7 years to 6.9 years were enrolled in the study. The median age of the children was 5.5 years, and there was an equal number of boys and girls. Nasal screening identified 17 (25%) *S. aureus *carriers including 8 (11.8%) CA methicillin-sensitive *S. aureus *(MSSA) carriers and 9 (13.2%) CA-MRSA carriers. Of the 9 children colonized with CA-MRSA (median age, 5.7 years), none exhibited any identified risk factor.

### Antibiotic susceptibility profiles of CA-MRSA colonization isolates

Antimicrobial susceptibility testing of the 9 CA-MRSA colonization isolates showed the following susceptibility rates: vancomycin (100%), gentamicin (88.9%), tetracycline (100%), clindamycin (0%), erythromycin (0%), trimethoprim/sulfamethoxazole (100%) and ciprofloxacin (100%). For CA-MSSA and CA-MRSA colonization isolates, the rates of resistance to erythromycin (50% and 100%, respectively) and clindamycin (0% and 100%, respectively) were significantly different. Moreover, the CA-MRSA isolates from children in the colonization-group were more likely to be susceptible to gentamicin, tetracycline, trimethoprim/sulfamethoxazole, and ciprofloxacin than the HA-MRSA isolates (Table 1). No significant differences were found between the 2 groups with respect to susceptibilities to either clindamycin or erythromycin.

**Table 1:** Antibiotic susceptibility of MRSA isolates from the kindergarten and hospitalized patients

	% of Resistant			
			
Antibiotic	Kindergarten Colonizers (N = 9)	Clinical Hospital Strains (N = 10)	95% CI*	P-value
Penicillin	100	100	NA/NM	NA/NM
Clindamycin	100	100	NA/NM	NA/NM
Erythromycin	100	100	NA/NM	NA/NM
Gentamicin	11.1	100	57.82–119.98	**0.001**
Vancomycin	0	0	NA/NM	NA/NM
Tetracycline	0	90	60.85–119.15	**0.001**
Trimethoprim/sulfamethoxazole	0	90	60.85–119.15	**0.001**
Ciprofloxacin	0	100	89.44–110.56	**<0.001**

*NA, not applicable; NM, not measured.

Of the 9 CA-MRSA colonization isolates tested using the double-disk diffusion method and PCR for detection of macrolide resistance genes, all had the MLS-constitutive (MLSc) phenotype and *ermB *gene.

### Molecular characterization of CA-MRSA colonization isolates

**Figure 1:**
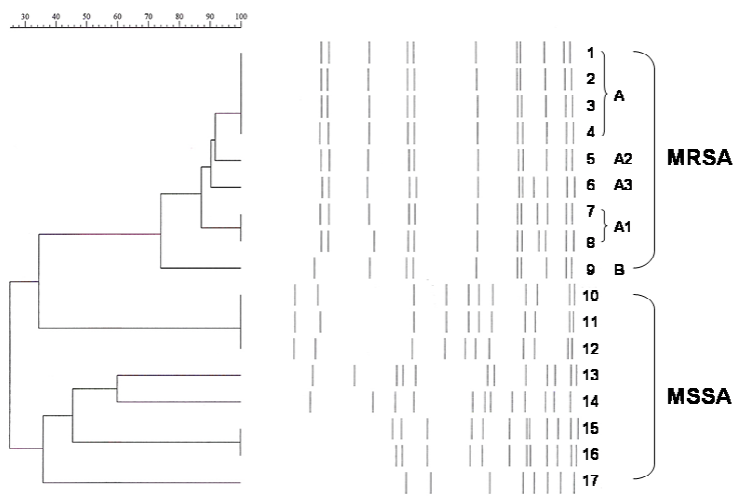
Schematic representation of PFGE pulsotypes of 17 study isolates (lanes 1–17) together with a dendrogram showing percent similarities of patterns.

All of the 9 CA-MRSA colonization isolates tested positive for the *mecA *gene and had the ST59 genetic background. The 17 *S. aureus *isolates collected for PFGE typing were categorized into 7 distinct PFGE patterns (Figure 1). The pulsotypes of all 9 CA-MRSA colonization isolates tested differed from those of the 8 MSSA colonization isolates. In addition, based on the interpretable phylogenetic tree, 1 set of CA-MRSA colonization isolates (type A, A_1_, A_2 _and A_3_) (8/9; 88.9%) appeared to be clustered, with homology percentages of >80%, while the other isolate from 1 colonized child was a distinct strain, type B. Two subjects with CA-MRSA were a sibling pair and their isolates belonged to strain type A. The 2 children colonized with strain type A_1 _were unrelated. Moreover, in addition to the 9 CA-MRSA colonization isolates, HA-MRSA isolates from clinical specimens collected from our hospitalized patients and matched by the geographical area and study period were also included in the PFGE analysis. The *Sma*I genomic fingerprints of the HA-MRSA isolates from these corresponding hospitalized patients were found to be totally different from those of the CA-MRSA nasal-colonization isolates (data not shown).

The results of SCC*mec *typing demonstrated that SCC*mec *type IV (100%) was the most-common type, nevertheless, staphylococcal toxin genes *lukS-PV *and *lukF-PV *were not present in any of the 9 CA-MRSA colonization isolates.

## Discussion

The *S. aureus *prevalence among children participating in this study was 25%, a value consistent with historical rates of *S. aureus *colonization [21]. Although various reports have suggested that the carriage of MRSA by persons without health care-associated risks has increased, significant heterogeneity among the study population existed [22]. The rate of CA-MRSA carriage of 13.2% in this study is within the reported range for children [23-25]. However, a recent study by Alfaro et al [26] found the highest reported rate (22%) of MRSA carriage in a group of South Texas children. This rate, which was higher than in the present study, was also found in one of the first known areas to experience the emergence and epidemic of CA-MRSA infections in children.

Review of antimicrobial susceptibility patterns in this study demonstrated that both clindamycin and erythromycin resistance were extraordinarily high among our CA-MRSA colonization isolates, which is in contrast with previous studies of CA-MRSA isolates from hospitalized children or adults, and colonized children [4,27,28]. Based on erythromycin resistance, *S. aureus *can be divided into 2 distinct phenotypes expressed either constitutively or inducibly [19]. In this study, the MLS_c _phenotype was more prevalent than MLS_i _phenotype in CA-MRSA colonization isolates, which is in agreement with previous studies [19,29,30]. In 2002, Almer et al [19] and also other investigators [29,31] reported *ermA *was the dominant *erm *gene present in their MRSA isolates and that the prevalence of *ermB *in *S. aureus *was less than 2%. Recently, SCC*mec *types II and III in HA-MRSA isolates were shown to contain *ermA *gene on a transposon (Tn*554*), which is responsible for inducible MLS resistance [32-34]. Moreover, in USA300 (ST8) CA-MRSA strains, constitutive MLS resistance was mediated by *ermC *gene that is typically located on a plasmid (pUSA03) [35,36]. In the present study, however, *ermB *was more widespread than *ermA *or *ermC *among CA-MRSA colonization isolates, which is in agreement with our recent findings in CA-MRSA isolates causing skin and soft-tissue infections [7]. In Taiwan, the widespread use of antimicrobials in the community may elicit some level of selective pressure which may account for the remarkably high incidence of clindamycin and erythromycin resistance among CA-MRSA isolates from colonized children [37,38].

In Europe, PVL genes have been associated with *S. aureus *isolates deriving from community-associated staphylococcal skin infections and also necrotizing pneumonia [18,39]. Recent evidence revealed that, although they do not share a common genetic lineage, PVL genes and the SCC*mec *IV allele are more prevalent among CA-MRSA strains from 3 different continents than among HA-MRSA strains globally [40]. In our colonized CA-MRSA isolates, SCC*mec *IV was indeed common, however, PVL genes were not found in any of these isolates. Since this observation is somewhat limited by the small number of isolates analyzed, persistent sampling from different parts of Taiwan and further molecular studies of such isolates are required to clarify this phenomenon. Although all 9 CA-MRSA colonization isolates were ST59, comparisons of PFGE patterns in this study indicated that 8 (88.9%) of the 9 isolates from this kindergarten were of a single clonal origin. The eight patterns were not indistinguishable by PFGE, but differed by 1 to 2 bands. Analysis of the results of this study suggest that 1 predominant CA-MRSA strain was circulating in the studied kindergarten and may be colonizing individuals not previously believed to be at risk.

## Conclusion

Our data suggest that the clonal spread of CA-MRSA was principally responsible for the high MRSA burden in this kindergarten. Of particular interest and importance is the future impact to the community or, indeed, elsewhere, should this common clone of CA-MRSA become more prevalent in Taiwan. Accordingly, much larger epidemiological studies pertaining to MRSA in the community are warranted to determine the scope of this emerging problem.

## Abbreviations used

CA-MRSA, community-acquired methicillin-resistant *Staphylococcus aureus*; HA-MRSA, healthcare-associated methicillin-resistant *Staphylococcus aureus*; MLS, macrolide-lincosamide-streptogramin; PFGE, pulsed-field gel electrophoresis; SCC*mec*, staphylococcal cassette chromosome *mec*; PCR, polymerase chain reaction; PVL, Panton-Valentine leukocidin; MSSA, methicillin-sensitive *S. aureus*.

## Competing interests

The author(s) declare that they have no competing interests.

## Authors' contributions

WTL conceived the study and designed it together with CCW. WTL conducted the experiments with contribution from WJL, MHT, JJL, SYL, MLC, and CCW. WTL, WJL, and MHT collected isolates. WTL drafted the article with contribution from JJL, MLC, and CCW.

## Pre-publication history

The pre-publication history for this paper can be accessed here:


